# Dots, Circles, and Deception: Radiological Diagnosis of an Unusual Mycetoma

**DOI:** 10.7759/cureus.103249

**Published:** 2026-02-08

**Authors:** Ria M Phulwani, Kajal Mitra, Suresh Phatak, Prashant Onkar, Mayank Rangari

**Affiliations:** 1 Department of Radiodiagnosis and Imaging, NKP Salve Institute of Medical Sciences and Research Centre, Nagpur, IND

**Keywords:** actinomycetoma, atypical presentation, dot-in-circle, eumycetoma, mycetoma, swelling

## Abstract

Mycetoma is a chronic granulomatous fungal or bacterial infection of the subcutaneous tissues, often with a characteristic clinical pattern, but in some patients, the presentation may be subtle or atypical, which can complicate diagnosis. Here, we report an unusual and rare presentation of mycetoma in a 26-year-old male who developed a chronic, progressively enlarging swelling over the leg. The lesion clinically resembled a soft tissue tumor, with no discharging sinuses present throughout the course of evaluation. Initial clinical impression favored a neoplastic or inflammatory etiology rather than an infective process. Ultrasonography and MRI demonstrated focal “dot-in-circle” appearances within the lesion, which supported the diagnosis of mycetoma in this case. The patient subsequently underwent surgical excision of the lesion, and postoperative histopathological examination confirmed the diagnosis of eumycotic mycetoma. This case underscores the importance of recognizing typical imaging features of mycetoma, particularly in the absence of classical clinical findings, to avoid diagnostic delay. Cross-sectional imaging plays a key role in improving diagnostic confidence in early or clinically ambiguous presentations.

## Introduction

Mycetoma (Madura foot), caused by fungi (eumycetoma) or filamentous bacteria (actinomycetoma), is a chronic granulomatous infection that usually presents with the classical triad of painless swelling, multiple sinus tracts, fistulas, and discharge of granules [1]. The condition initially presents as a localized subcutaneous swelling, yet may extend into adjacent tissue planes, affecting the skin, deeper layers, and ultimately the osseous structures [2]. The foot is the most frequently affected site, much more than areas such as the lower leg, hands, head, neck, chest, shoulders, or arms. The condition occurs predominantly in men, typically from ages 20 to 50 years. On histological examination, the disease is marked by the presence of “grains,” clusters of fungal hyphae or bacterial elements, embedded in micro-abscesses surrounded by granulomatous fibrous tissue [3]. It was first recognized in 1846 in the Indian region of Madura, which is why it later acquired the label “Madura foot” [4]. Endemic in Africa, Mexico, India, and the East, eumycetoma tends to occur in regions with low rainfall, commonly caused by *Madurella grisea*, *Pseudallescheria boydii*, and *Madurella mycetoma*, whereas actinomycetoma is more frequently found in areas with high rainfall, among which *Streptomyces madurae* and *Nippostrongylus brasiliensis* are the most common [5].

Mycetoma presentations that deviate from its classical clinical profile are infrequently encountered and may be easily overlooked. This case is interesting, being a rare presentation at an uncommon site with atypical clinical examination findings that closely resembled a soft-tissue tumor and hence escaped correct clinical recognition. Documentation of these unusual presentations is important for broadening clinical suspicion, emphasizing the diagnostic value of radiological assessment, and promoting inclusion of mycetoma as a potential diagnosis in patients presenting with long-standing subcutaneous swellings at atypical sites.

## Case presentation

**Figure 1:**
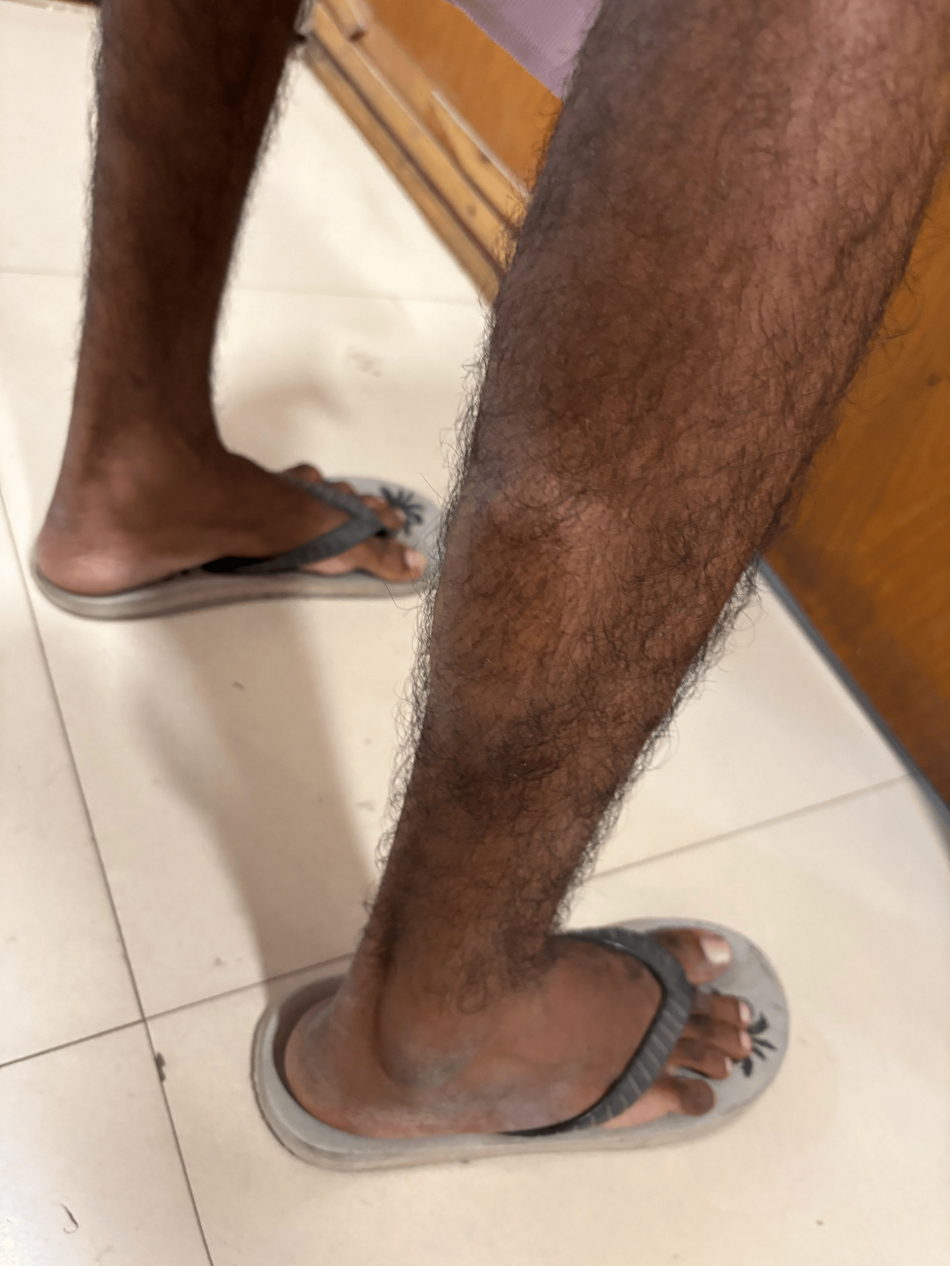
Clinical photograph showing swelling of the right leg at presentation.

A 26-year-old male presented with a swelling over the lateral aspect of the right leg (Figure 1) since May 2016. The swelling was initially small and gradually increased to its present size, as mentioned below. The patient reported associated pain for the past six months. There was no history of fever or trauma at the site of the swelling. On examination, a swelling measuring approximately 2 × 5.4 × 7.5 cm was noted over the lateral aspect of the right leg. It was firm to hard in consistency, with a few areas of surface nodularity, and showed no local rise of temperature. Laboratory parameters were within normal limits (hemoglobin: 15.6 grams per deciliter, total leukocyte count: 8000 cells per microliter, platelets: 301,000 cells per microliter, and C-reactive protein: 68 milligrams per liter)

**Figure 2:**
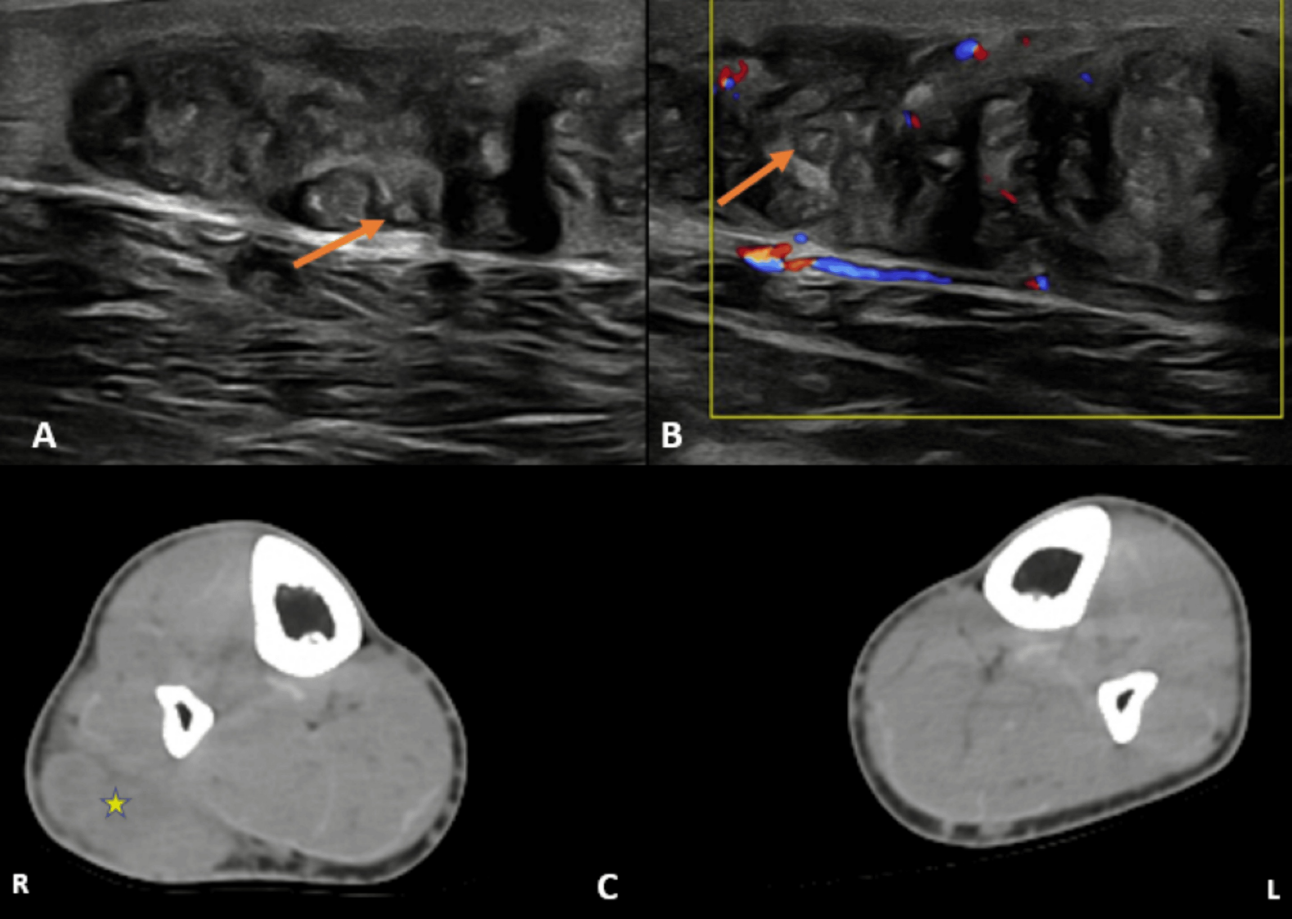
On ultrasound, a well-defined, heterogeneous, predominantly hypoechoic lesion of size 1.8 x 5.1 x 7.1 cm was noted in the subcutaneous plane in the mid-calf region, at the postero-lateral aspect of the right leg. (A) A few round hypoechoic areas with hyperechoic central foci were observed (orange arrow). (B) On color Doppler, it showed focal areas of vascularity. (C) On further investigation, non-contrast axial CT showed a well-defined, lobulated, heterogeneous, predominantly isoechoic soft tissue mass lesion at the postero-lateral aspect of the right leg, in the subcutaneous plane with mild adjacent fat stranding (yellow star). No evidence of any surrounding fluid attenuation or discharging sinuses was noted.

Imaging revealed a well-defined, lobulated, heterogeneous soft-tissue lesion located in the subcutaneous plane of the postero-lateral aspect of the right mid-calf. On ultrasonography (Figures 2A, 2B), the lesion was predominantly hypoechoic with a few round hypoechoic areas containing central hyperechoic foci. Non-contrast CT (Figure 2C) demonstrated a well-defined, lobulated, heterogeneous soft-tissue mass with mild adjacent fat stranding.

**Figure 3:**
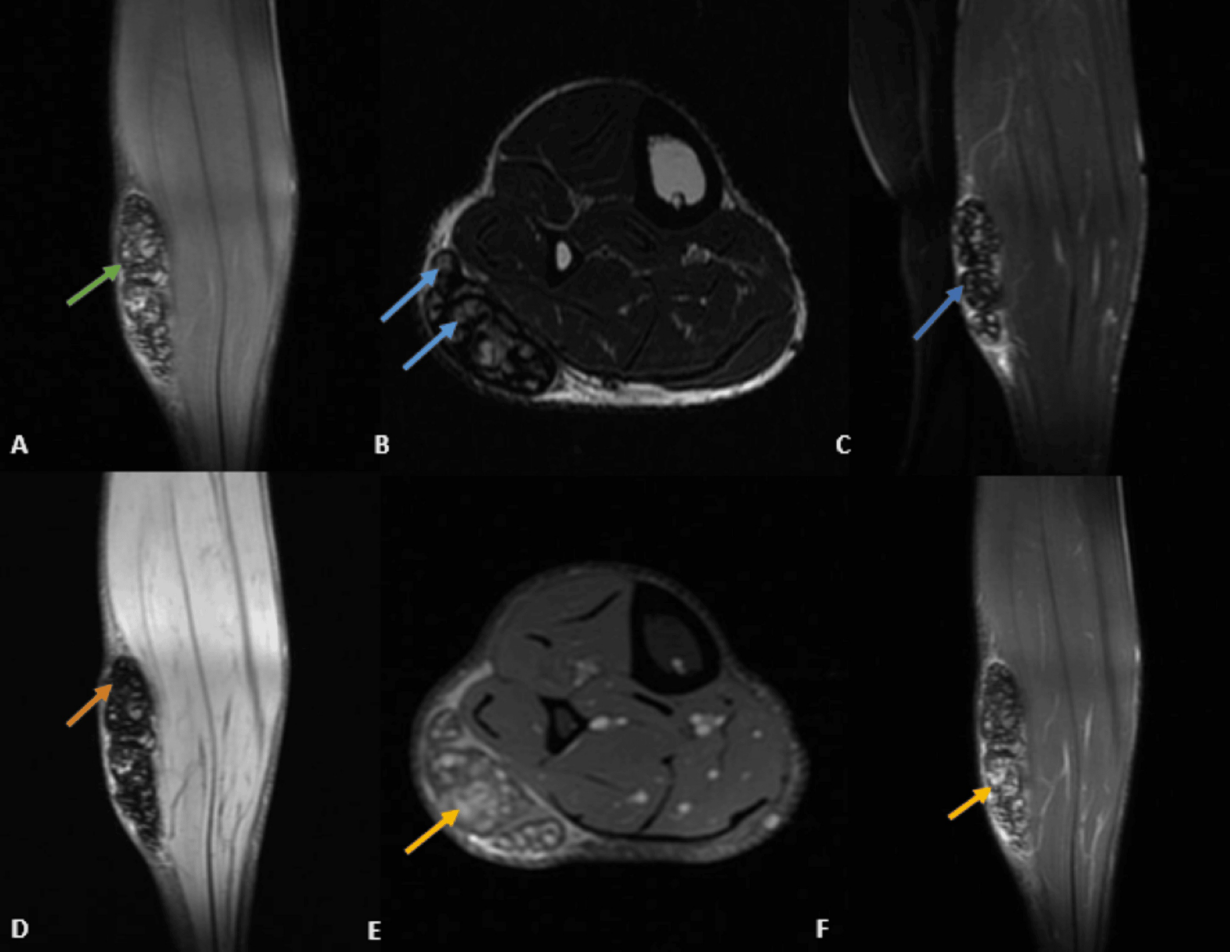
On further MRI, a large, well-defined, ovoid, lobulated, heterogeneous altered signal intensity lesion measuring 3.9 x 2.2 x 9.8 cm was noted in the subcutaneous plane of the postero-lateral aspect of the right leg, overlying the fibularis longus and soleus muscles (mid-shaft region). (A) On T1 (coronal), the lesion (green arrow) was isotense to hypointense compared with the skeletal muscles. (B, C) On T2 (axial) and STIR (coronal), it appeared heterogeneous with multiple internal hypointense septa/reticulations. Few areas of the dot in the circle were observed (blue arrows). (D) On MERGE (coronal), ovoid areas of blooming are noted within (orange arrow). Mild edema of the adjacent subcutaneous tissue was noted. (E, F) On post-contrast images (axial and coronal), it shows heterogeneous enhancement (yellow arrows). No evidence of any underlying muscular extension, abscess, or discharging sinuses was observed. MERGE, multiple echo recombined gradient echo; STIR, short tau inversion recovery.

On MRI (Figures 3A-3F), it appeared heterogeneous in signal intensity, measuring up to 9.8 cm in craniocaudal extent, overlying the fibularis longus and soleus muscles, and heterogeneous post-contrast enhancement; however, no evidence of muscular invasion, abscess formation, or discharging sinuses was noted.

Treatment and outcome

**Figure 4:**
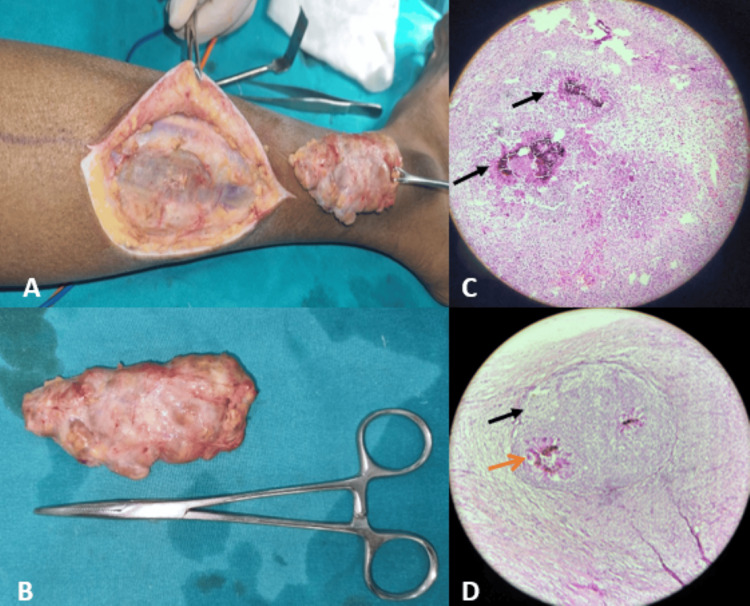
(A, B) Intraoperative images. (C) Histopathological analysis of the postoperative specimen confirmed the imaging-based diagnosis of subcutaneous eumycotic mycetoma. Few granulomas show colonies of septate fungal hyphae with brown pigment (black arrows). (D) Black arrow: granuloma and orange arrow: fungal colonies

The patient was initiated on antifungal therapy with oral itraconazole at a dose of 200 mg twice daily for a duration of one month; however, there was no clinical response on follow-up. Consequently, surgical excision was performed (Figures 4A, 4B). The surgery was uneventful, and the soft tissue lesion was completely removed and sent for histopathological analysis. The histopathological report was suggestive of subcutaneous eumycotic mycetoma (Figures 4C, 4D).

**Figure 5:**
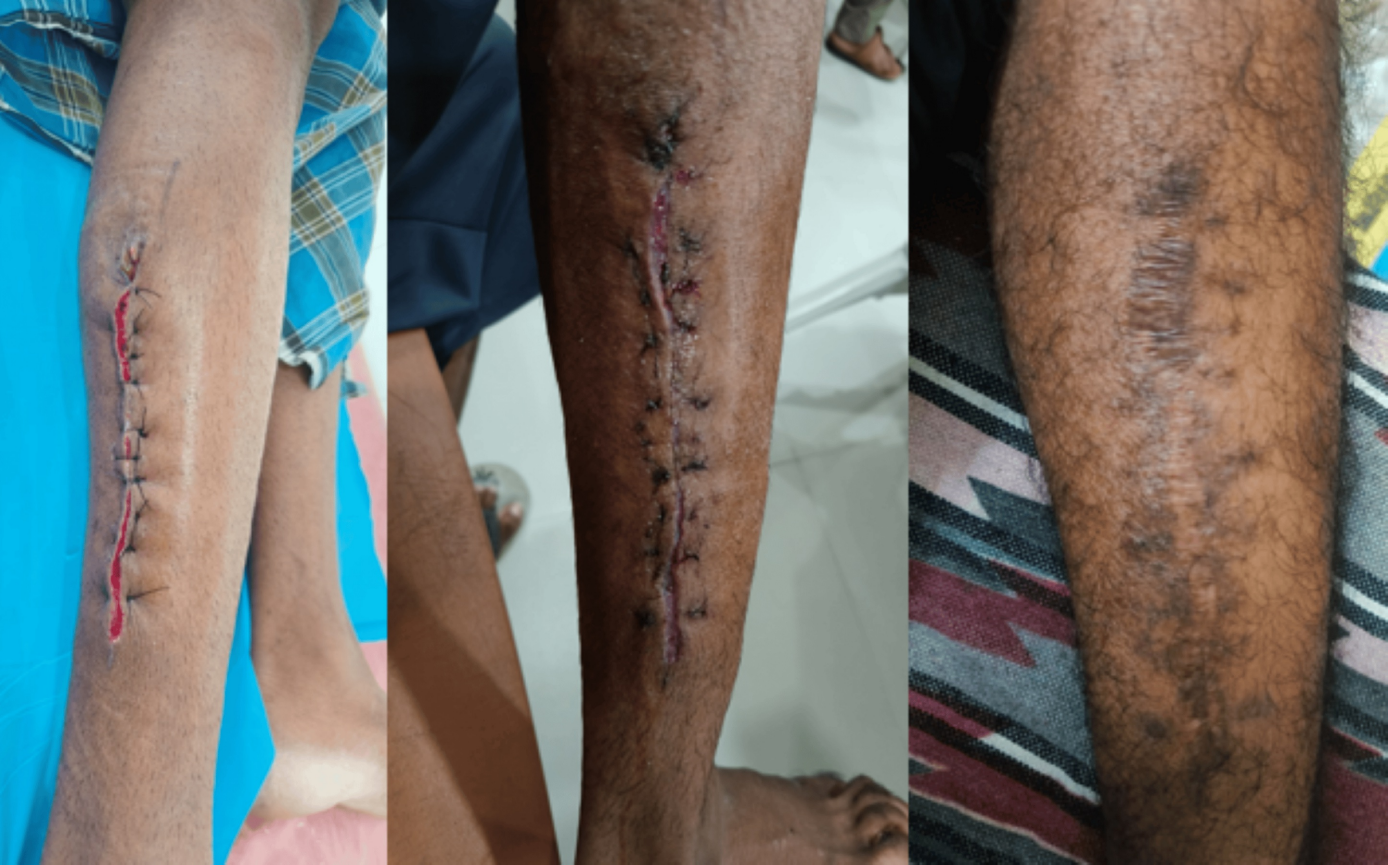
Serial follow-up images

 The patient remains asymptomatic on follow-up with no pain, swelling, or sinus discharge (Figure 5).

## Discussion

Madura foot, or mycetoma, represents a persistent granulomatous disease of the dermal and subdermal tissues, caused by fungal organisms in eumycetoma or by *Actinomyces* species in actinomycetoma [6]. The causative organism is generally believed to enter the body when the skin is pierced by a sharp object such as a thorn, resulting in direct inoculation [7].

Clinically, patients usually develop painless nodules beneath the skin along with fistulous tracts that may release purulent material. Although the condition tends to progress slowly, it can eventually lead to abscesses, draining sinuses, osteomyelitis, and additional fistula formation [8], ultimately causing significant deformity/disability if left untreated.

Antifungal therapy is effective in almost 90% of affected individuals; however, cases involving sites other than the foot or those caused by non-fungal organisms generally have a poorer outlook and often require surgical management [7]. Staining techniques such as Gram stain, periodic acid-Schiff, lactophenol cotton blue, and methenamine silver are valuable for distinguishing actinomycetoma from eumycetoma [9].

Diagnosing the condition early, before grains or sinus tracts appear, is challenging. Although definitive diagnosis can usually be obtained through biopsy showing characteristic features or by staining and culturing discharge from the lesion, these procedures are time-intensive and may be difficult to perform, particularly when dealing with fastidious organisms. Radiographs may appear normal or may show soft-tissue swelling, bone sclerosis, cortical scalloping, expansion, periosteal reactions, cavitations, or osteoporosis [10]. Unlike bacterial osteomyelitis, mycetoma typically involves the bone from the outside inward [11], and certain features help differentiate eumycetoma from actinomycetoma: the former produces fewer but larger bone cavities (≥1 cm), whereas the latter creates numerous small cavities, giving a moth-eaten pattern [8].

Ultrasound findings, described by Fahal et al. [12], show echogenic foci representing grains: sharp echoes in eumycetoma and finer, settling echoes in actinomycetoma. The ultrasonography “dot-in-circle” pattern mirrors the MRI sign [12].

CT offers clearer visualization of these bone changes compared with plain films [13]. Early MRI reports described low T1 and T2 signals due to susceptibility effects from the grains [14]. The characteristic “dot-in-circle” sign, tiny low-signal dots within hyperintense rounded lesions [15], corresponds histologically to granulomas (bright areas), surrounding fibrosis, and central grains (dark foci). This sign has since been reported repeatedly but may be misinterpreted, as in one case, mistaken for a hemangioma. Rice bodies in tuberculous arthritis can also mimic the “dots” [16].

## Conclusions

This case highlights an atypical presentation of mycetoma occurring in the leg without sinus formation or bone involvement, features that can make early recognition challenging. The identification of the few areas of dot-in-circle, which is a hallmark of eumycetoma, on ultrasound, with further investigation on CT and MRI, proved crucial in establishing the diagnosis at an unusual site. This underscores the value of cross-sectional imaging in detecting mycetoma even when classical clinical features are absent, allowing timely and appropriate management.
